# First evaluation of the performance of portable IGRA, QIAreach® QuantiFERON®-TB in intermediate TB incidence setting

**DOI:** 10.1371/journal.pone.0279882

**Published:** 2023-02-10

**Authors:** Zirwatul Adilah Aziz, Noorliza Mohamad Noordin, Wan Mazlina Wan Mohd, Mohd Amin Kasim

**Affiliations:** National Public Health Laboratory, Ministry of Health, Selangor, Malaysia; U.S. Food and Drug Administration, UNITED STATES

## Abstract

Diagnosis and treatment of tuberculosis infection (TBI) are the core elements of tuberculosis elimination. Interferon gamma release assays have advantages over the tuberculin skin test, although their implementation in low-resource settings is challenging. The performance of a novel digital lateral flow assay QIAreach^®^ QuantiFERON^®^-TB (QIAreach QFT) against the QuantiFERON^®^-TB Gold Plus (QFT-Plus) assay was evaluated in an intermediate incidence setting (Malaysia) according to the manufacturer’s instructions. Individuals aged 4–82 years, who were candidates for TB infection screening for contact investigation were prospectively recruited. On 196 samples, the QIAreach-QFT showed a positive percent agreement (sensitivity) was 96.5% (CI 87.9–99.6%), a negative percent agreement (specificity) 94.2% (CI 88.4% to 97.6%) and an overall percentage of agreement was 94.9% (95% CI 90.6–97.6%) with a Cohen’s κ of 0,88. Out of 196, 5.6% (11/196) samples gave an error result on QIAreach-QFT and 4.1% (8/196) samples gave indeterminate result on QFT-plus. The TTR for QIAreach QFT positive samples varied from 210–1200 seconds (20 min) and significantly correlated with IFN-γ level of QFT-Plus. QIAreach QFT could be considered an accurate and reliable point-of-need test to diagnose TB infection helping to achieve the WHO End TB programme goals even in decentralised settings where laboratory expertise and infrastructure may be limited.

## Introduction

The World Health Organization (WHO) reports that tuberculosis (TB) still represents one of the major killers, causing around 1.5 million deaths worldwide annually [1–4]. TB still is, globally, among the 10 commonest causes of preventable death, as both TB and TB infection can be successfully treated.

The latest WHO estimates show that many gains achieved in recent years reversed in 2020 due to COVID-19 pandemic with 1.5 million lives lost due to TB and significantly less people enrolled on TB preventive therapy in 2020 [1, 5–10].

WHO has recently emphasized the public health importance of managing TB infection and included the integrated patient-centred care and prevention approach under Pillar 1 of the End TB Strategy [2, 11, 12]. Furthermore, while diagnosis and treatment of infectious TB patients are the essence of TB control, diagnosis and management of TB infection are the core activities to pursue TB elimination [8, 9]. Screening for TB infection and TB preventive treatment become even more important in the COVID-19 era when many TB services worldwide have been severely disrupted [1, 13].

We have two families of tests to diagnose TB infection [12, 14], tuberculin skin tests (TST) and interferon-γ (IFN-γ) release assays (IGRAs). TST, more than 100 years old, is a skin test in which a given amount of tuberculin (purified protein derivative-PPD) from mycobacterial culture is intradermally injected to generate a delayed type of hypersensitivity reaction, producing an induration, which is measured in millimetres [14]. Novel skin-based tests that seem to elicit more specific immune responses to *Mycobacterium tuberculosis* are under development [15]. However, all skin tests are associated with the same operational challenges as TST: lack of quality control, subjective measurement of test results and challenges in data recording, training needed for standardized administration and reading of results and, finally, two patient clinical visits required for tuberculin administration and follow-up reading [15, 16]. IGRAs have been developed more recently (13–18). They are in vitro assays able to measure the blood levels of IFN-γ released by T lymphocytes stimulated with antigenic peptides of *M*. *tuberculosis* [14, 17–22].

So far, WHO has endorsed three IGRA tests, T-SPOT^Ⓡ^.*TB* (Oxford Immunotec, Abingdon, UK), Beijing Wantai’s TBIGRA (Wantai), and QuantiFERON^Ⓡ^-TB Gold Plus (QFT, QIAGEN, Hilden, Germany), able to measure IFN-γ released by both CD4 and CD8 T cells [23–28].

Although WHO-endorsed IGRA tests have advantages over TST, both require professional expertise and considerable laboratory infrastructure thus not qualifying yet as point-of-care tests. This is clearly a factor limiting their use in resource-limited settings [14, 29, 30]

In Malaysia, we use either tuberculin skin test (TST) and interferon-gamma release assays (IGRA) for the diagnosis of latent TB infection in adult and children.

A potential step forward to provide a “field friendly” solution in high burden TB settings which combines the measured benefits of IGRA technology with a point-of-care test that can be used outside traditional laboratory settings is represented by the QIAreach^**®**^ QuantiFERON-TB (QIAreach QFT) assay [29, 31, 32].

QIAreach QFT leverage the same technology as the TB2 tube of QFT-Plus, is portable, simple to perform and requires only 1 ml of blood from each patient [14, 29, 31–33].

So far three studies have compared the new QIAreach QFT test against the established (FDA[Food and Drug Administration]-approved and CE [European Community]-marked) QuantiFERON^Ⓡ^-TB Gold Plus test in detecting TB infection, both conducted in low incidence settings (Milan, Japan and USA, respectively) [29, 31, 33].

This study aims to evaluate QIAreach^®^ QFT diagnostic performance in an intermediate TB burden country using the QuantiFERON^®^-TB Gold Plus ELISA (QFT-Plus) as a reference standard.

## Materials and methods

### Study population

This prospective trial was conducted between April 2021 and June 2021 at the the National Public Health Laboratory, Sungai Buloh, Malaysia. This study was approved by Medical Research and Ethics Committee (MREC), Ministry of Health. Only those individuals who agreed to participate and signed a written informed consent form were included in the study. For the respondent 18 years and above, the respondents must personally agree before performing any specific procedures, and the participation is voluntary. For respondents less than 18 years, the parents must sign the informed consent form. Nevertheless, before respondents participate in this research study, the researchers will explain the study, and respondents have a chance to ask questions. After the respondents are adequately satisfied and wish to participate in this study, the respondent must sign informed consent form. The respondent is free to withdraw from the study at any time for any reason and will not affect any medical care or any benefits to which respondents are entitled. Demographic data collected from patients included age, gender as well as reason for testing (Table 1) parameter Individuals aged 4–82 years, who were candidates for TB routine infection screening test for contact investigation were prospectively recruited. All enrolled participants were interviewed by the attending physician and underwent a chest radiography (CXR). For those with radiographic anomalies suggestive of TB, further evaluation was performed using sputum acid-fast bacilli smear and culture to rule out active TB or lung disease due to nontuberculous mycobacteria.

**Table 1:** Characteristics of study participants.

Characteristics	Individual screened (n = 196)
Age, years; median (IQR)	15.5 (25.75–41.25)
Sex, male; n (%)	73(37.2%)
Contact of TB case; n (%)	163 (83,2%)
Contact of MDR-TB case; n (%)	67 (16,8%)

IQR: Interquartile range, n = number, MDR-TB = Multidrug-resistant TB

None of all subjects undergoing to QIAreach-QFT testing had active TB. For all participants, 1ml aliquots of blood were drawn directly into four blood collections tubes and used to run both QFT-Plus and QIAreach QFT tests. Ethical approval for this study was obtained from the Medical Research and Ethics Committee (MREC), Ministry of Health Malaysia (Approval No.: NMRR 17-315-34830).

### QFT-Plus assay

QFT-Plus assays were carried out according to the manufacturer’s instructions (QIAGEN GmbH, Hilden, Germany) [34]. Venous blood was collected from the patients into the following four blood collections tubes: Nil (negative) Control, Mitogen (positive) Control, TB1 Antigen, and TB2 Antigen tubes (0.8ml-1.2 ml each). Within 16 h after blood collection the tubes were incubated at 37°C for 16–24 h. After incubation, the tubes were centrifuged at 3000×*g* for 15 min. Plasma supernatants were harvested and then processed immediately or stored at 4°C until enzyme-linked immunosorbent assay (ELISA) was performed. IFN-γ levels were quantified for QFT-Plus samples with 4-point standard curves. Results were reported as TB antigen minus nil values. The optical density of each well was measured by reading in the ELISA reader using the “QuantiFERON-TB Gold Plus Software”. The results were interpreted as Positive, Negative, or Indeterminate according to the Instructions For Use.

### QIAreach QFT assay

The QIAreach QFT assay was performed according to the manufacturer’s instructions [29, 31], and used in combination with the QIAreach Software (prototype versionx64-1.1.12.0) installed on a computer running the Microsoft Windows operating system.

Prior to running the assay, the eHub was powered on. The eStick comes packaged with a process tube that contains dried nanoparticle conjugate. Immediately prior to use, the eStick and Processing tube were removed from their packaging and the eStick was inserted into the eHub.

Assay Diluent Buffer (150 μL) was added to the Processing Tube to resuspend the dried nanoparticle conjugate, followed by addition of 150 μL of plasma specimen from the patient TB2 tube into the Processing Tube. The plasma sample was mixed with the resuspended nanoparticle conjugate in the Processing Tube with the pipette, then150 μL of the mixed sample was transferred from the Processing Tube into the sample port of the eStick connected to the eHub port. The sample was detected automatically in the eStick, and after test completion the assay results (positive or negative and Time To Results) were displayed on both the computer and the eHub.

### Statistical analysis

Qualitatively data were expressed as IFN-γ concentration IU/ml or Time To Results (TTR) and analyzed using Microsoft Excel application spreadsheet software. The concordance between the QFT-Plus and the QIAreach QFT assay results was assessed using kappa coefficients and was interpreted according to the Landis and Koch classification [35]. Consequently, the Cohen’s kappa test results were classified as follows: a score of < 0.2 indicated slight agreement; 0.21–0.40, fair agreement; 0.41–0.60, moderate agreement; 0.61–0.80, substantial agreement; and 0.81–1, almost perfect or perfect agreement. The overall percent agreement (OPA) was determined using QFT-Plus as the reference method. The positive percent agreement (PPA; sensitivity) and negative percent agreement (NPA; specificity) were calculated along with the corresponding exact two-sided 95% Confidence Interval (CI) using QFT-Plus as a reference. Linear regression analysis was performed to examine the relationship between the QIAreach QFT TTR (in seconds) and the QFT-Plus IFN-γ levels (in IU/mL).

## Results

QIAreach QFT and QFT-Plus were blinded performed in 196 patients, 163 (83.2%) being contacts of drug-susceptible TB and 33 (16.8%) of MDR-TB patients with median (IQR) age of 31 years (25.25–41.75); 73/196 (37.2%) were males. No patient was undergoing dialysis or was immunocompromised.

Out of 196 samples tested, 5.6% (11/196) samples gave an error result on QIAreach-QFT and 4.1% (8/196 of whom one children immunosuppressed and 7 contact of TB case > 9 years old without any underlying medical condition) samples gave indeterminate result on QFT-plus. These 19 specimens have been excluded from further analysis. As reported in Table 2, the QIAreach-QFT showed a positive percent agreement (sensitivity) was 96.5% (CI 87.9–99.6%), a negative percent agreement (specificity) 94.2% (CI 88.4% to 97.6%) and an overall percentage of agreement was 94.9% (95% CI 90.6–97.6%) with a Cohen’s κ of 0,88. Out of 9 discordant samples, 7 samples were positive by QIAreach-QFT alone and 2 samples were positive to QFT-plus alone.

**Table 2:** Diagnostic performance of QIAreach QFT assay using QFT-plus as a reference standard.

	Frequency	Agreement	Upper 95% CI	Lower 95% CI
OPA	169/178	94.9%	90.6%	97.6%
PPA	55/57	96.5%	87.9%	99.6%
NPA	114/121	94.2%	88.4%	97.6%
Cohen’s κ score	0.88			

OPA: Overall Percent Agreement, PPA: Positive Percent Agreement, NPA: Negative Percent Agreement

**Fig 1:**
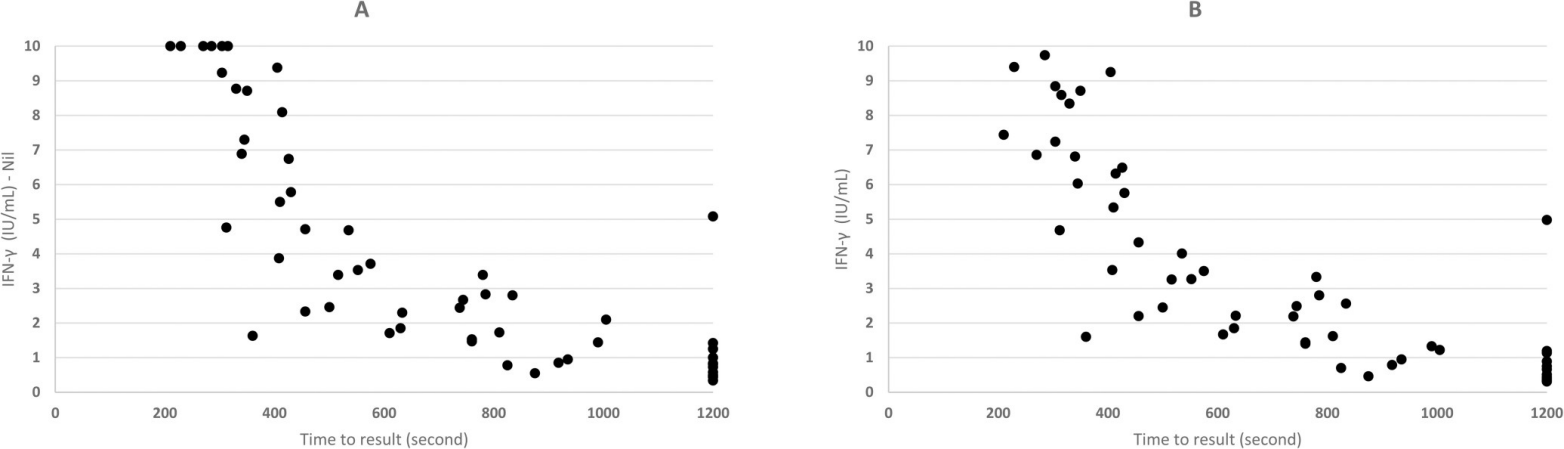
Distribution plot of IFN-γ values (uncorrected and corrected TB2 values) versus TTR for positive samples (n = 55). A- uncorrected values. B–corrected values.

In thirteen QFT-Plus positive specimens, both TB1-Nil and TB2-Nil value were <1 IU/ml and all returned a positive QIAreach QFT result. The TTR for QIAreach QFT positive samples varied from 210 second (3,5 minutes) to 1200 seconds (20 min). Fig 1 shows the distribution plot of QFT-Plus IFN- γ concentrations (IU/ml) in TB2-Nil (corrected) and TB2 (uncorrected) tube values versus TTR (in seconds) for QIAreach QFT positive samples. The linear regression analysis demonstrated a strong negative correlation between TTR and IFN-γ levels in corrected and uncorrected TB2 with a correlation coefficient R = -0.7984 (p < 0.001) and R = -0.7961 (p < 0.001) respectively.

## Discussion

Our study aimed at evaluating the performance of the QIAreach QFT assay using the globally established QFT-Plus IGRA as a reference method.

This study represents the first performance evaluation performed in an intermediate TB burden setting. The study results demonstrated an excellent level of agreement as well as high sensitivity and specificity of QIAreach QFT compared to QFT-Plus when implemented in routine settings in Malaysia, confirming previous results achieved in Milan, Japan and USA [29, 31, 33]: OPA, PPA (sensitivity) and NPA (specificity) exceeding 94% [36].

The QIAreach QFT overall error rate was 5.6% (11/196), five errors were related to eStick functioning, while six were run or user workflow errors. The high concordance between QIAreach QFT and QFT-Plus results, specifically in QFT-Plus patients with TB1 and/or TB2 antigen values < 1 IU/mL, supports comparable sensitivity of the QIAreach QFT detection system to the QFT-Plus ELISA. Similarly to QFT-Plus with value close to the assay cut-off (0.35 IU/ml), QIAreach QFT may potentially have some variability of results with a TTR close to 1200 seconds. In our study nine samples had discrepant results between QIAreach QFT and QFT-Plus. Discordant results may be influenced by a number of factors including but not limited to borderline IU/mL values near the cut-off, the absence of a Nil background subtraction in the QIAreach QFT test, sample viscosity and color affecting lateral flow functionality, but nevertheless, a high agreement was observed between the novel QIAreach QFT detection system and the established QFT-Plus ELISA.

Fig 1 shows the IFN-γ level of the corrected and uncorrected TB2 values of QFT-Plus plotted against the QIAreach QFT TTR. We found a statistically significant relationship between levels of corrected and uncorrected IFN-γ in TB2 and TTR with a correlation index of R = -0.7984 (p < 0.001) and R = -0.7961 (p < 0.001) respectively. Similar findings were reported by Fukushima et al. suggesting a high correlation between IFN-γ level and TTR [29].

QIAreach QFT showed several benefits for low resource, high burden testing markets. The simple workflow and use of an integrated lateral flow test with highly sensitive detection capabilities instead of ELISA translates into less laboratory infrastructure and minimal required training, key benefits for the implementation in resource-limited settings. The volume of blood is remarkably reduced when compared to the conventional IGRA assay. QIAreach QFT requires 1 ml of blood compared to the 4 ml needed to perform the QFT-Plus ELISA. This is of particular relevance in young children where high volume is not always easy to collect. Optional software is also available with the QIAreach QFT assay to assist with report generation and connectivity through LIMS. Finally, QIAreach QFT can run eight samples independently, providing a readout in ≤ 20 minutes after a simple assay set up of less than 1 minute. With each of the eight eHub ports operating independently, the use of multiple eHubs provides testing scalability options. This translates into a total of 88–96 samples/days which make this assay even more suitable for mass screening.

In view of the urgency to apply the WHO End TB Strategy [11] and pursue TB Elimination [8] it is important to strengthen the prevention measures and to adequately diagnose and treat TB infection [2, 12].

The advantages of the IGRA tests, in terms of sensitivity, specificity, need for a single visit, simple interpretation of results (not subject to inter- and intra-reader variation of the induration reading in millimeters), no influence by previous Bacillus Calmette-Guérin (BCG) vaccination(s) are well known (12). In a recent meta-analysis published the QFT-Plus assay had a pooled sensitivity of 94% for active TB patients and a pooled specificity of 96% for healthy low risk individuals [37]. However, the previous generation of IGRAs cannot be considered point-of-care tests as they are based on the ELISA technology and need substantial laboratory facility resources and equipment. Clinical performance of novel skin tests based on specific *M*.*tuberculosis* antigens developed over the last decade is yet to be fully established and they share the same operational challenges as TST [15]. These include need of training for administration of the test and reading of results, quality control challenges, lack of objectivity, need for a return visit for reading the results and cold chain for storage and transportation of antigen preparation.

New technologies like QIAreach QFT may overcome several of these logistic and performance challenges. The QIAreach QFT is simple to implement in any setting (including mobile stations, remote operation) and the workflow requires minimal laboratory expertise. The rapid turnaround time is an additional element making this assay a valuable tool for the decentralization of TB infection diagnosis with moderate test throughput.

The study has several limitations, all related with its preliminary nature: it was conducted in a single laboratory (the reference one for Malaysia) on a sample of individuals who were contacts of drug susceptible of MDR-TB patients, so that evidence on other important target groups is not yet available; the sample size with regards to statistical power was not calculated.

Larger studies are necessary to further evaluate the potentiality of this assay in different settings (particularly in low income, high burden countries) and different groups of individuals, and different groups of individuals, as well as data on precision and costs analysis to create sufficient evidence and therefore validate preliminary results in the field [38]. Increasing the accessibility to TB infection management is, in fact, a priority for the years to come.

## Supporting information

S1 Data (XLSX)
